# Influence of Finishing Process Parameters of HDF Boards on Selected Properties of Coatings in Modern UV Lines and Their Relation to Energy Consumption

**DOI:** 10.3390/ma17225393

**Published:** 2024-11-05

**Authors:** Maciej Tokarczyk, Barbara Lis, Tomasz Krystofiak

**Affiliations:** 1 Department of Wood Science and Thermal Techniques, Faculty of Forestry and Wood Technology, Poznan University of Life Sciences, Wojska Polskiego 28, 60-627 Poznan, Poland or maciej.tokarczyk@up.poznan.pl (M.T.); barbara.lis@up.poznan.pl (B.L.); 2 BORNE-FURNITURE Company, Złotego Smoka Str. 23, 66-400 Gorzów Wlkp., Poland

**Keywords:** surface, UV lacquer system, energy, UV curing process, topography, contact angle, Wenzel coefficient

## Abstract

This study analyzes the influence of energy generated by emitters on the adhesive properties of varnish coatings in multilayer UV systems. The experimental material, in the form of a cell board finished with UV varnish products, was prepared on a prototype line under the conditions of Borne Furniture in Gorzów Wielkopolski. The roughness and wettability were measured using a OneAttension tensiometer integrated with a topographic module, taking into account the Wenzel coefficient. The adhesion of the examined systems was verified using the PositiTest AT-A automatic pull-off device. Energy consumption by the prototype production line was compared to the standard line, utilizing mercury emitters and mercury emitters with added gallium. Energy consumption was calculated for selected variants. The influence of the Wenzel coefficient on the wettability angle was observed. Significant differences between contact angles (CA and CAc) were noted for coatings formed with sealers (stages I and II). The largest discrepancies, reaching up to 30 degrees, were recorded at the lowest UVA and UVV doses of 26 mJ/cm^2^. In adhesion tests, values below 1 MPa were obtained. Insufficient energy doses in the curing process of UV systems led to delamination between the coatings. Five variants were selected where delamination within the substrate predominated (˃90% A) and were characterized by the lowest energy consumption in the processes. Topographic images helped identify the presence of various surface microstructures at different stages of the production cycle. The greatest energy savings, up to 50%, were achieved in stages III and IV of the technological process.

## 1. Introduction

Various types of wood and wood-based materials are used as substrates in the production of furniture and interior furnishings. Their surfaces can be finished in numerous ways, depending on the desired aesthetic–decorative and functional properties [1]. A crucial step influencing the final characteristics of finished products is the selection of specific raw materials, which dictates the application of appropriate production technologies. The most common method of protecting wood and wood-based materials in furniture production and interior furnishings is the use of lacquering technology [2]. Liquid materials can be applied in various ways, ranging from the simplest methods to fully automated processes within comprehensive production lines. Before choosing a specific technology, it is essential to define the requirements that finished products should meet. This involves determining numerous material and technological parameters related to the type of lacquers and finished elements, operating conditions, desired resistance, aesthetic appeal, and application speed, which correlates with production efficiency. The specification of requirements during the production preparation stage and the proper execution of individual operations will determine the final properties of the finished surfaces. All factors must be considered together, as they interact through feedback mechanisms [3,4,5].

The surface refinement process influences the utility value of furniture, encompassing both appearance and durability. In addition to ensuring high product quality, manufacturers should also consider aspects related to the eco-efficiency of their production processes. These are now fundamental features of developmental technologies. Therefore, during the selection of technological processes necessary for product manufacturing, decision making must involve solutions that encompass not only quality parameters but also environmental criteria. These actions are supported by entrepreneurs as they also consider the economic profitability of the technologies and products. The use of surface refinement technologies involving lacquers is inherently associated with the generation of environmental impacts. Although these impacts cannot be entirely eliminated, they can be minimized, thereby significantly preventing negative consequences [6,7]. Reducing these impacts enhances the ecological and energy efficiency of technologies, ultimately translating into the financial performance of enterprises. With this in mind, environmentally friendly products and the implementation of innovative solutions are gaining increasing importance. In line with this trend, environmentally sustainable lacquers, referred to as “green coatings”, are being widely introduced into industrial practice [6,7,8,9]. These coatings ensure a safe working environment during the production, application, and use of the coatings. These aspects are particularly important for products used in finishing items that are used in residential spaces. To achieve these solutions, solvent-based products with high solid contents, waterborne products, powder coatings, bio-based materials, and UV, IST, or EBC curing techniques are utilized [6,8,10,11,12,13]. To meet increasing demands, new formulations of lacquers are being developed, alongside the implementation of highly automated and robotized production systems. Significant progress has been made in thin-layer applications using rollers, which allow for increased material efficiency. Various roller designs have been developed, including solutions related to the number of rollers, direction, and speed of operation [4,10]. Accumulated experience has enabled the application of lacquers with varying viscosities, ranging from 20 s to semi-fluid mass consistencies, and reducing the amount of applied lacquer. Roller coaters are often combined with printing technology. These technologies are further integrated with radiation curing methods, in which products undergo UV-induced polymerization based on free-radical reaction mechanisms. Despite the initially high investment costs, this system is considered highly efficient and forward looking due to the significant increase in the curing speed of products, thereby enhancing production efficiency, reducing energy costs, lowering VOC emissions, and achieving high resistance in the resulting coatings [11,12,14,15,16,17,18,19,20,21,22,23,24,25].

Lacquering lines used for these products vary in the number of basic machines and devices, as well as in their design solutions. Manufacturers must adapt the production process to the specific lacquer product to obtain finished products with the best possible functional properties. Additionally, appropriate design and technical solutions for production lines can ensure lower energy consumption. One energy-saving initiative in UV technology is the use of UV LED lamps, which offer many advantages over traditional radiation sources [26,27,28]. These lamps operate based on electroluminescent diodes, which consume significantly less electricity compared to conventional lamps that require large energy input to excite mercury vapor. They feature low-heat emissions without the need for so-called cold mirrors, ensuring that the generated UV radiation is effectively used for curing, whereas in conventional lamps, a large amount of energy is converted into heat. Another advantage is their immediate readiness for operation upon switching on. This allows for turning off the lamps during production breaks, reducing the energy demand. This also translates into a longer service life [26,27,28,29,30,31,32]. Additional energy savings can be achieved by selecting curing power to ensure optimal coating parameters while rationally using energy [32]. The energy efficiency delivered to the surface can be regulated by adjusting the distance of the lamps from the elements passing beneath them or the conveyor speed, which determines the exposure time of the lacquered product [33,34]. Monitoring the spectral distribution of radiation emitted by UV lamps, thus verifying their efficiency, is carried out using radiometers. By utilizing these devices, energy consumption can be effectively managed to reduce costs. For conventional lamps, energy savings are estimated to range from 50% to 80% [35,36]. However, it should be noted that energy efficiency is influenced by a range of factors present at different production stages, which must be thoroughly analyzed. UV LED technology has tremendous potential for energy optimization while maintaining high product quality.

This article focuses on analyzing the impact of UV curing process parameters on the development of the selected properties of coatings, such as roughness, contact angle, adhesion, and surface topography. To demonstrate the practical implementation of LED lamps in multilayer UV systems, a comparison was made of the energy consumption in the curing process using mercury lamps and mercury–gallium lamps. Based on the obtained results, the curing parameters were identified, which achieved optimal energy reduction while maintaining the high quality of the final products.

## 2. Materials and Methods

### 2.1. Substrate and Coating Products

**Figure 1:**
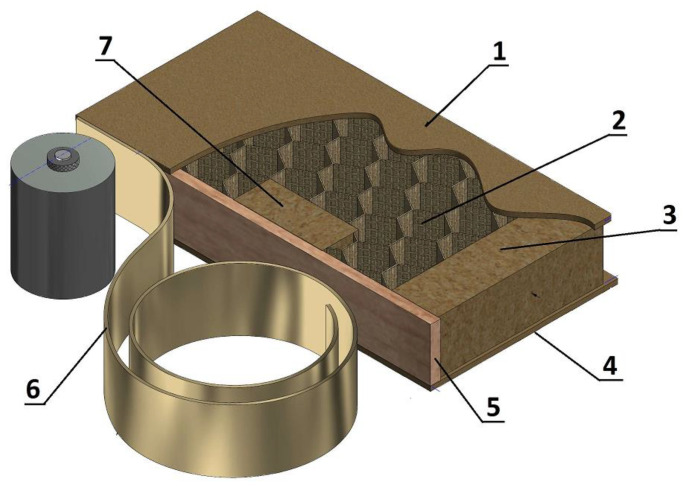
Lightweight wood-based honeycomb furniture panels with discreetly located strengthening blocks. Element description: (**1**) top panel (HDF); (**2**) honeycomb; (**3**) cross bars (optional); (**4**) bottom panel (HDF); (**5**) support edge; (**6**) edge band (ABS); (**7**) strengthening blocks [37].

In this study, a lightweight cellular panel with HDF external facings and a cellular core reinforced with blocks measuring 100 × 60 cm (Figure 1) was employed [37]. The samples were fabricated using an innovative method that has been patented under the title “Method for Manufacturing a Cellular Plate and a Cellular Plate Manufactured by This Method” (PL234055B1) [38]. Currently, approval is pending for an EU-scale patent for a related invention titled “Method for Manufacturing an Ultra-Light Cellular Board with Blocks and an Ultra-Light Cellular Board with Blocks” (PL443573) [39]. The ultra-lightweight cellular board, which is the subject of the invention, consists of three layers. Between the two outer layers made of HDF boards with a density of 850 ± 30 kg/m³, there is a core characterized by discretely arranged reinforcing rectangular elements, referred to as blocks, made of particleboard with a density of 630 ± 30 kg/m³. The space between the blocks is filled with a honeycomb paper structure (paper weight 135 ± 5 g/m^2^), with cell sizes of 18 ± 2 mm, providing additional internal support.

The blocks were initially fixed to the lower HDF plate using EVA hot melt adhesive, and the entire structure was pressed in a hydraulic shelf press at a temperature of 60 ± 5 °C and a pressure of 1.2 kg/cm^2^. To glue the whole construction, a water dispersion of polyvinyl acetate (PVAC) with D2 resistance class was used (solid content: 48 ± 1%; viscosity acc. to the Brookfield RV: 13,000 ± 2000 mPa·s).

**Figure 2:**
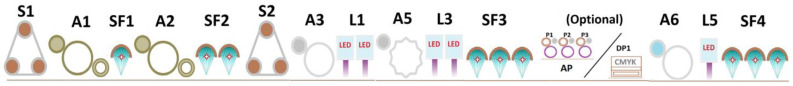
Selected schematic diagram for finishing the board furniture components with a roller method and analog printing technology. Process description: (**A1**) sealer 1–50 g/m^2^; (**A2**) sealer 2–25 g/m^2^; (**A3**) basecoat 1–12 g/m^2^; (**A5**) basecoat 2–30 g/m^2^; (**A6**) topcoat 1–5 g/m^2^; (**S1**,**S2**) sanding; (**AP**: **P1**,**P2**,**P3**) analog printing machine; (**DP1**) digital printing machine; (**L1**,**L3**,**L5**) LED lamp 395 nm 12 W/cm^2^; (**SF1**,**SF2**,**SF3**,**SF4**) Super-Focus Mercury Lamp 120 W/cm.

The application process of multilayer coating systems with analog printing was carried out at a line feed speed of 40 m/min according to the general concept presented in Figure 2. The concept is based on result of research and development works of project POIR.01.01.01-00-1235/17, Development of technology for finishing the surface of wood materials using selected energy-saving sources electromagnetic radiation at advanced speeds and varnish systems characterized by increased resistance. Properties of lacquer products used for research was given in the Table 1.

The samples were prepared under production conditions on the test line. The air temperature was 25 ± 5 °C, and the relative humidity (RH) was 40 ± 10%. The liquid materials were conditioned at a temperature of 25 ± 5 °C. Applicator types (manufactured by Cefla, Imola, Italy), UV modules (manufactured by Cefla, Imola, Italy) and their parameters, and other components (transport systems manufactured by Cefla, Imola, Italy; sanding machines manufactured by Karl Heesemann Maschinenfabrik GmbH & Co., Bad Oeynhausen, Germany) of the test line are presented with labelling in Table 2. The board furniture elements prepared at the different stages were transported to the laboratory, cut into samples, and left in the air-conditioned laboratory at 23 ± 2 °C, 50 ± 5% relative humidity.

### 2.2. Research Method

#### 2.2.1. Measurements of Energy

**Figure 3:**
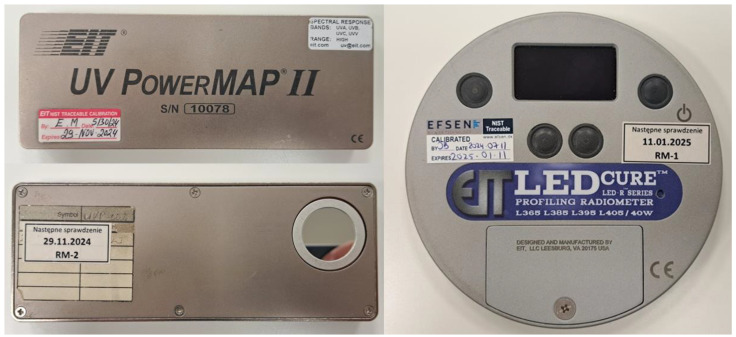
Electronic radiometer by EIT^®^ and EIT2.0^®^.

The measurements of energy generated by the lamps were conducted on a prototype production line using radiometers placed on a conveyor moving under the emitters, recording the curing parameters. The assessment of the emitted energy and, thus, the technical condition of the Hg emitters was performed using a Power Map 2 radiometer (EIT^®^, made in the Leesburg, VA, USA), while the LED modules were evaluated using the LED CURE 4CH model PL-4C-P40 (Figure 3), manufactured by EIT2.0^®^, (Leesburg, VA, USA). The former allows for precise radiation intensity measurements across various ultraviolet ranges (UVA 320–390 nm, UVB 280–320 nm, UVC 250–260 nm, UVV 390–450 nm). Meanwhile, the LED CURE 4CH radiometer enables the determination of radiation intensity and energy density at specific LED wavelengths L365 (340–392 nm), L385 (360–412 nm), L395 nm (370–422 nm), L405 (380–422 nm), with max power 40 W/m^2^.

#### 2.2.2. Roughness

The roughness measurements were conducted using an optical method by replicating the surface profile through a structured light technique known as phase-shifted fringe projection. For this purpose, a topography module accounting for roughness, integrated with the OneAttension Theta tensiometer (Biolin Scientific AB, Gothenburg, Sweden), was used. The roughness parameters were calculated based on the three-dimensional shape of the object, in accordance with the procedure described in the Biolin company manual [40,41]. Among the recorded parameters, two indicators, Ra and Rz, were selected. Table 3 presents the parameters related to the measurement system.

#### 2.2.3. Contact Angle

The contact angle measurements were performed using the optical tensiometer OneAttension Theta (Biolin Scientific AB, Sweden) by placing a 3.5 µL drop of distilled water on the tested surface. The measurement was conducted over 60 s, during which the shape of the droplet was recorded by a camera. Subsequently, the image was analyzed using specialized OneAttension software (One Attention version 4.2.1), which calculated the contact angle (CA) and the corrected value with the Wenzel factor (CAc). The wettability assessment was carried out in the same location on the sample where the roughness measurement was performed, in accordance with the procedure described in the Biolin company manual [40,41].

#### 2.2.4. Adhesion Strength of Coatings

**Figure 4:**
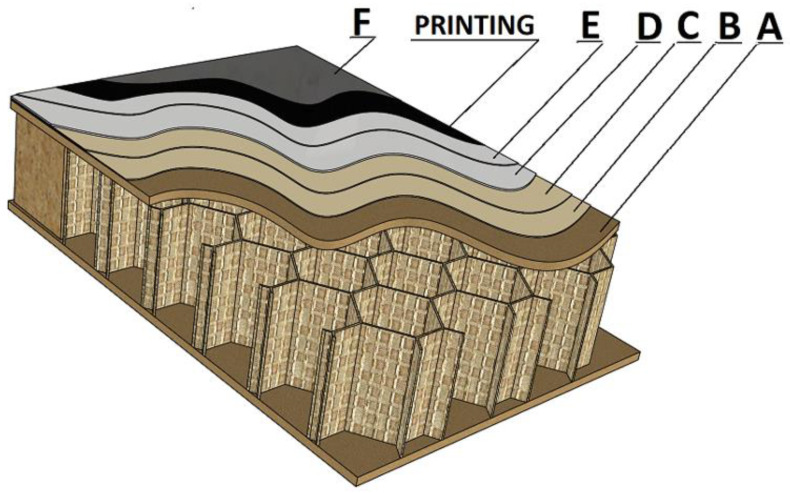
Sample with labelled layers.

The study was conducted according to the PN EN ISO 4624 standard using an automatic PositiTest AT-A device (DeFelsko Corporation, Ogdensburg, NY, USA) [42]. Before starting the test, five incisions with a diameter of 20 mm were made on the surface of the samples using a circumferential cutter. Measurement stamps were then attached using JOWAT 690.00 silane-epoxy adhesive (JOWAT SE, Detmold, Germany). The samples were conditioned under RT conditions (temperature 20 ± 5 °C and RH 65 ± 5%). After 168 h, the detachment of the samples was performed. After each test, the delamination patterns caused by destructive loads were visually assessed according to the evaluation scale provided in Table 4 and Figure 4.

#### 2.2.5. Surface Topography

The surface topography was measured using a topography module integrated with the OneAttension Theta tensiometer (Biolin Scientific AB, Sweden). During the study, a digital camera recorded fringe patterns, which were used to reconstruct the surface shape of the sample. Both 2D and 3D measurements were conducted, in accordance with the procedure described in the Biolin company manual [40,41]. The parameters of the measurement system are provided in Table 3.

## 3. Results

### 3.1. Roughness

**Figure 5:**
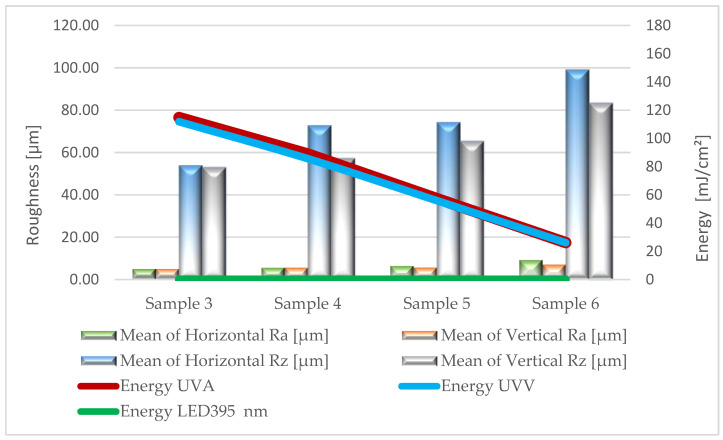
Formation of the values of the Ra and Rz parameters depending on the dose of delivered energy for coatings formed in Stage I.

**Figure 6:**
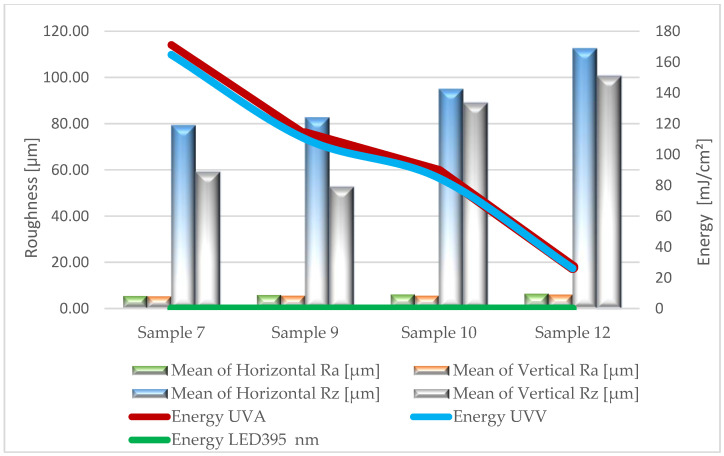
Formation of the values of the Ra and Rz parameters depending on the dose of delivered energy for coatings formed in Stage II.

**Figure 7:**
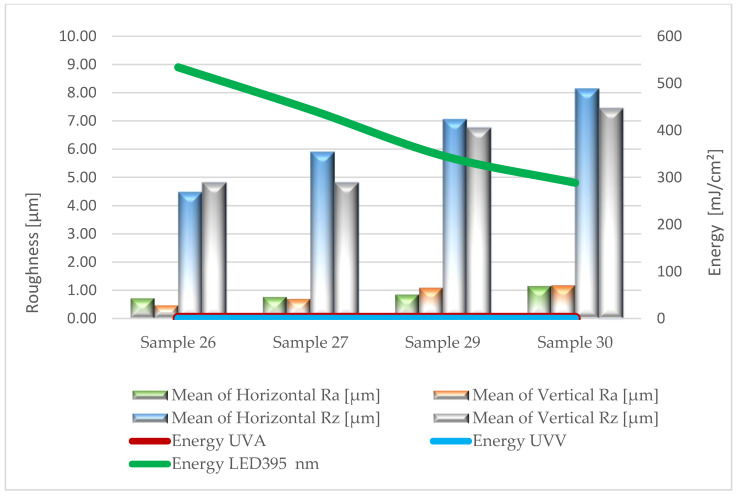
Formation of the values of the Ra and Rz parameters depending on the dose of delivered energy for coatings formed in Stage III.

**Figure 8:**
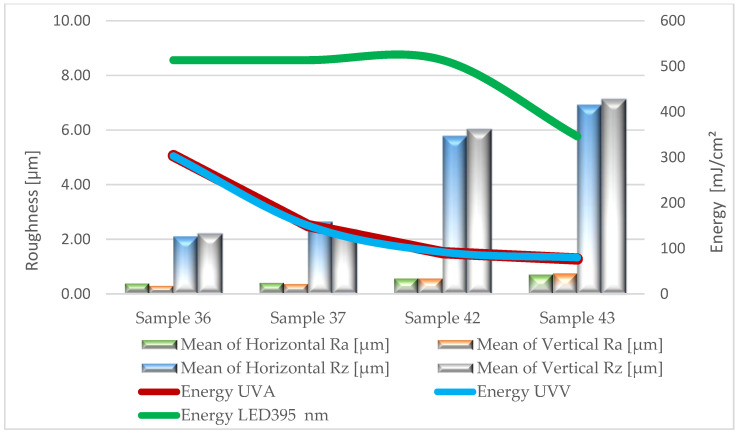
Formation of the values of the Ra and Rz parameters depending on the dose of delivered energy for coatings formed in Stage IV.

**Figure 9:**
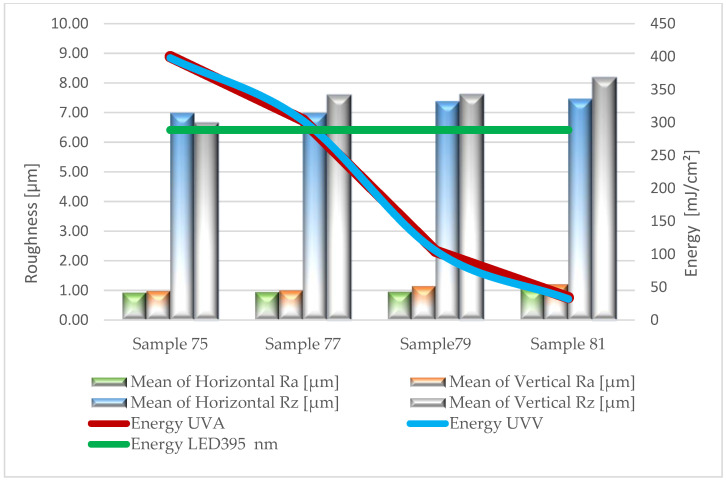
Formation of the values of the Ra and Rz parameters depending on the dose of delivered energy for coatings formed in Stage V.

Based on the results of surface roughness tests, Figure 5, Figure 6, Figure 7, Figure 8 and Figure 9 present the trend of changes in the values of the Ra and Rz parameters for individual variants hardened with different doses of energy distributed by radiators.

From the analysis (based on ANOVA analysis) of the presented dependencies, it follows that at almost each stage of production, as the energy density decreases, the roughness of the coatings increases. For the topcoat stage, the impact of energy density on roughness cannot be determined definitively (*p*-value 0.224–0.322). The studies did not show a clear trend in the changes of Ra and Rz parameters depending on the measurement direction. These observations apply to all variants, regardless of the type of lacquer product and the amount applied. This is particularly important when using multilayer UV lacquer systems in industrial conditions. Significantly higher values were recorded on the surface of sealers serving as primer layers. Undoubtedly, the substrate, in the form of HDF board, influenced the obtained results.

After applying the first sealer, the product spread and filled the surface irregularities resulting from previous sanding. This stage contributed to a certain replication of the surface shape of the board. For this group of tested variants, the Ra parameter ranged from 5.2 to 9.4 µm, while Rz ranged from 52.0 to 99.1 µm. In the initial phase of the technological process, this is a favorable phenomenon from the point of view of the interaction between the sealer and the substrate. High roughness provides better adhesion for the next applied lacquer layer. In the case of applying a second sealer layer, a significant development of the obtained coating surface was also noted. The Ra parameter ranged from 5.4 to 6.3 µm, while Rz ranged from 52.6 to 112.6 µm. Similar to the first layer, this promotes “mechanical adhesion”, according to which adhesion increases with increasing roughness. It should be noted that a larger roughness profile will significantly impact the consumption of lacquer products, especially filling materials. Free space (so-called dead volume) is created in the profile’s recesses, which must be filled to achieve a uniform coating thickness. This space is not considered when measuring coating thickness, so more lacquer products must be applied [43].

The application of the primer lacquer caused the surface to smooth out, reflected in the significant decrease in Ra parameters to the level of 0.5–1.2 µm and Rz to the values of 4.8–7.4 µm. The next layer of primer lacquer further reduced roughness (Ra = 0.3–0.8 µm; Rz = 2.1–7.1 µm). The increased smoothness of the hardened primer lacquer layer was influenced by their different physicochemical properties, which reduced viscosity. This improved the flowability and wettability of the substrate. Conversely, in the final stage of the technological process, after applying the top lacquer, an increase in the evaluated parameters was again observed, oscillating within the range of Ra 0.9–1.2 µm and Rz 6.7–8.2 µm. This may be a result of the degassing of the foamed lacquer material, the presence of matting agents, and slight shrinkage occurring during the curing process of the product.

Similar observations were made in a publication [44]. The research undertaken focused on the impact of selected technological parameters (number of layers, type of lacquer product, application amount, type of rollers, type of UV emitters, energy density supplied to the curing process, and production line speed) on the adhesion of lacquer coatings formed in the roller coating process. The surface roughness values obtained in this study confirmed that interlayer systems exhibit lower roughness values than the final coatings. Furthermore, it was shown that the measured Ra parameter values are smaller than Rz. However, as a result of using different surface roughness measurement techniques, different discrepancies between these indicators were noted. The values measured by the contact profilometer were lower than those obtained with the optical method. The results largely confirm the findings of Hazir and Koca, who, when analyzing Lebanese cedar (*Cedrus libani*) and black pine (*Pinus nigra*), achieved higher values using the non-contact method [45]. These results also align with the research by Henke, which concerned the effect of different abrasive belt configurations and conveyor speeds on the surface roughness of HDF boards [46].

### 3.2. Contact Angle

**Figure 10:**
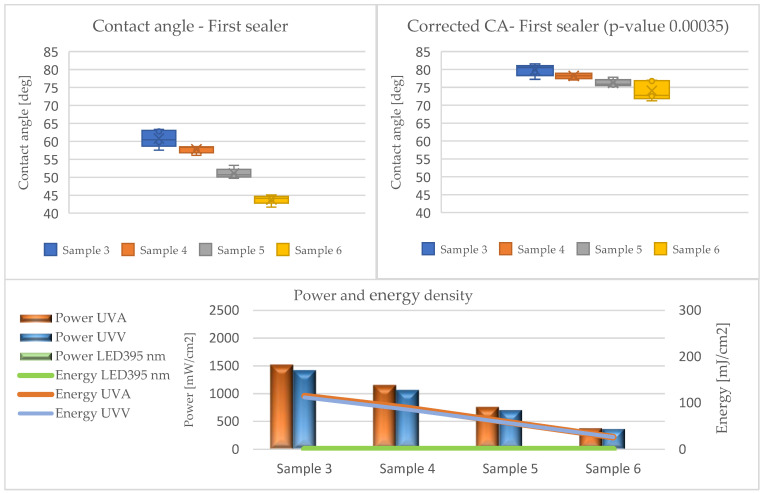
Formation of contact angle depending on power and energy density after applying the first sealer (Stage I).

**Figure 11:**
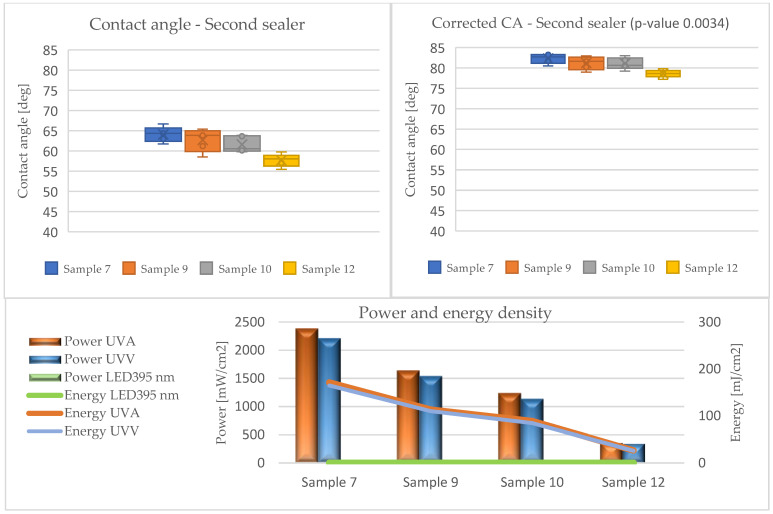
Formation of contact angle depending on power and energy density after applying the second sealer (Stage II).

**Figure 12:**
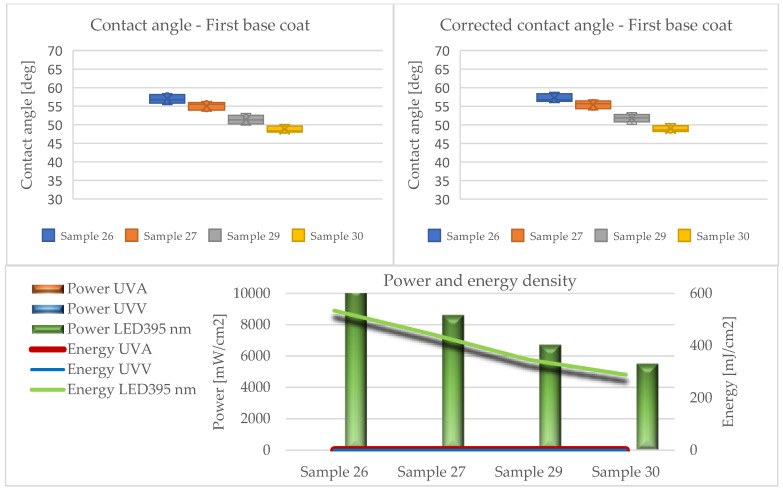
Formation of contact angle depending on power and energy density after applying the basecoat (Stage III).

**Figure 13:**
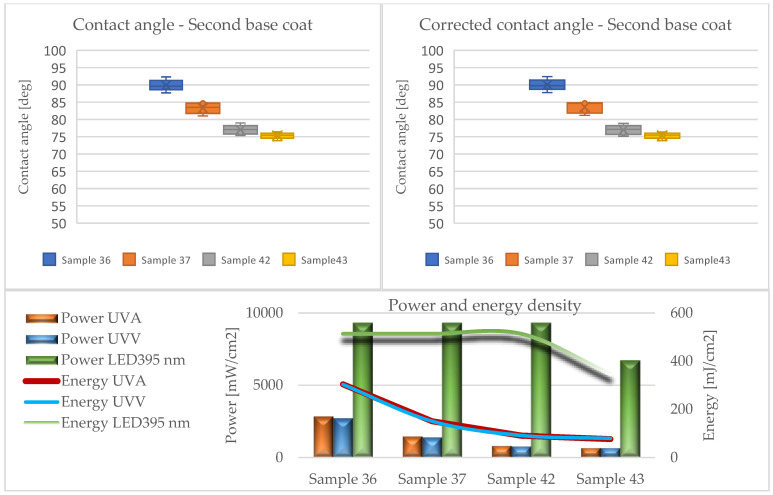
Formation of contact angle depending on power and energy density after applying the second basecoat (Stage IV).

**Figure 14:**
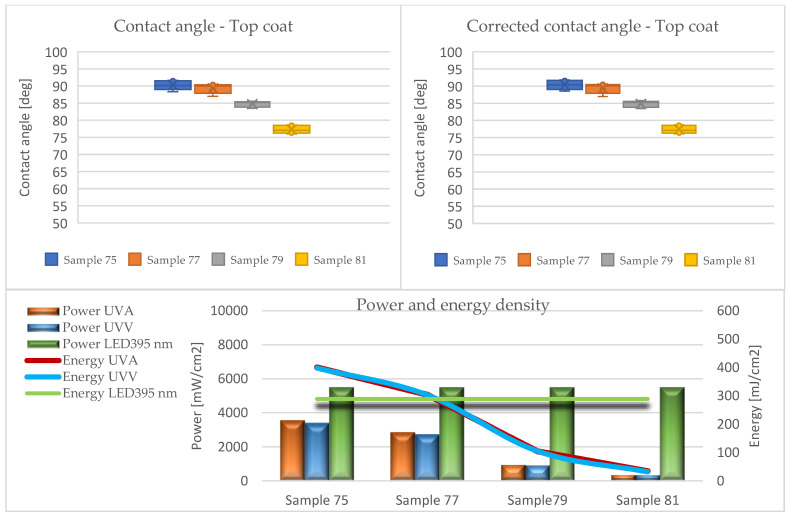
Formation of contact angle depending on power and energy density after applying the topcoat (Stage V).

The results of the contact angle tests in the form of average values without (CA) and with the Wenzel correction factor (CAc) are illustrated in the graphs (Figure 10, Figure 11, Figure 12, Figure 13 and Figure 14).

Analyzing the presented data (based on ANOVA analysis), it was determined that the dose of energy used for curing UV products influences the wetting process. Our previous research confirmed that the energy density supplied had the greatest influence on the wetting angle, with the power of the emitters directly affecting this factor. This indicated that process optimization should focus primarily on adjusting the emitter power and exposure time [44].

At all stages of the technological process, a decrease in the contact angle was observed with the reduction in energy density. This effect was most pronounced in the first stage, where the largest quantities of lacquer product were used in the entire process, and initial gelling occurred. Conversely, after applying the primer and curing it with LED lamps (Stage III), a decrease in the contact angle was observed compared to the layers formed from fillers. The obtained values were the lowest among all tested systems throughout the process, cured exclusively with LED radiators. The use of Hg lamps (stage IV) contributed to surface curing. The resulting contact angle values were close to those of the topcoats but not as high due to the application of 30 g/m^2^.

The influence of the surface’s geometric structure on the formation of this parameter was also observed. These interactions are explained, among other things, by the Wenzel roughness factor, according to which an increase in roughness leads to increased wettability [47,48]. Considering the impact of the Wenzel factor, significant differences between the angles (CA and CAc) were observed in the case of coatings formed from sealers (Stages I and II). The greatest discrepancies were noted at the lowest energy doses of UVA 26 mJ/cm^2^ and UVV 26 mJ/cm^2^ for Sample 6, reaching up to 30 degrees. This confirms the necessity of considering the Wenzel correction factor when assessing the wettability of HDF boards.

In the remaining variants, the differences oscillated around 20 degrees. This was due to the increased roughness of the formed layers, resulting from the interaction of the substrate surface on the formation of coating irregularities and the rheological properties of the sealers. The fillers were characterized by a high content of filling substances and high viscosity, leading to lower flowability, which, when applied to the substrate, caused larger irregularities.

In the subsequent stages, the used lacquer products exhibited lower viscosity, favorably affecting their spread on the HDF board. This significantly reduced the roughness of the cured layers, making the impact of irregularities on the contact angle minimal. It is assumed that if the roughness parameter Ra < 0.5 μm, it does not significantly influence the formation of the contact angle [48]. According to this assumption, only systems 36 and 37 met this criterion in the conducted studies. Nonetheless, for other variants, no large discrepancies between the CA and CAc angles were noted.

### 3.3. Adhesion Strength

In the adhesion tests of the individual layers, numerical values below 1 MPa were obtained. These values did not provide a definitive answer as to which of the tested systems could be considered superior. It was only through the damaged images that differences in the evaluated variants became apparent, resulting from the impact of the energy dose on the interlayer adhesion of the coatings. Given this, Table 5 highlights the systems exhibiting the most favorable adhesion (marked in green) along with the lowest energy density supplied to the process.

On the surfaces of the selected systems, delaminations in the substrate were predominant. Out of the five trials conducted, at least three showed damage exclusively in the substrate (100% A). In the remaining samples, delamination in the HDF board was recorded above 80%. An exception was sample 42, where delamination was observed at 30% A and 75% A/B. This indicates that the weakest link in the tested systems during the adhesion test was the HDF board. In other variants, problems with interlayer adhesion were observed, reflecting the type of delamination occurring between the individual coatings. This suggests inadequate curing (either over-curing or under-curing of the lacquer layer).

For Stage III, the use of only the LED lamp with the lowest energy dose at UVV 346 mJ/m^2^ resulted in C/D-type damage (system 30). Similarly, for variant 43 (Stage IV), UVA energy 26 mJ/m^2^ and UVV 27 mJ/m^2^ led to adhesion issues (primarily D/E delamination). On the other hand, poor properties for printing were exhibited by systems with curing above UVV 513 mJ/m^2^ and surface curing UVA 150 mJ/m^2^ UVV 150 mJ/m^2^ (systems 36, 37), which showed clear delamination of type 100E/Printing. These are the substrates showing negative properties as a base for printing. After applying the topcoat, Print/F type delamination was noted, indicating insufficient curing (system 81). This variant may be recommended as an interlayer but not as a final finish.

Our previous research confirmed the correct selection of lacquer products, including sealers, basecoats, and topcoats, as well as the appropriate technological parameters for the analyzed systems [44]. The expected outcome was to prepare variants where the weakest link in adhesion tests using the pull-off method would be cohesive failures within the substrate, rather than in the lacquer coatings or between them. The applied products were cured using various types of UV emitters, which allowed for the selection of optimal solutions for each stage of the process. In addition to mercury lamps, systems with the addition of gallium, known for their high efficiency and broad radiation spectrum, were also used, along with UV LED emitters. Additionally, modules equipped with systems for concentrating the UV radiation beam were applied, allowing focused energy to be directed onto the surface, thus increasing the curing efficiency. As a result, the tested systems exhibited excellent adhesion, as evidenced by delamination within the HDF board.

The results of the adhesion tests highlight the need to select appropriate lamp power, monitor the UV curing process in industrial conditions, and demonstrate the necessity of developing the number and power of innovative LED emitters.

### 3.4. Estimation of Topography

**Figure 15:**
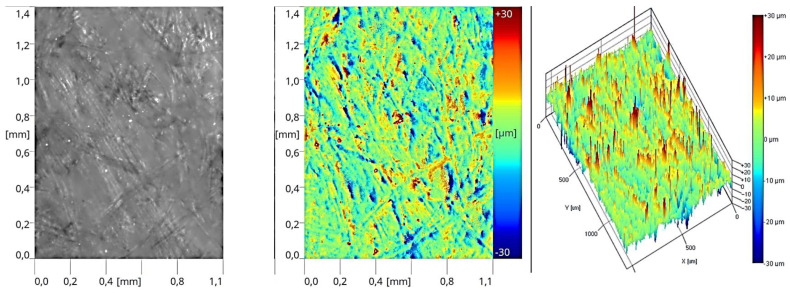
Surface image of the coating formed in Stage I (one layer of sealer).

The final assessment of the tested variants involved verifying the surface morphology of the applied layers during the printing process. Based on the results of the studies, images were presented that depicted the topography of the coatings that exhibited the most favorable adhesive properties among the tested systems. The obtained images revealed the presence of varying surface microstructures at different stages of production. These correlate with the roughness parameter results. After performing the initial operation, which involved applying 50 g/m^2^ of sealer, a distinct reflection of the substrate surface was observed (Figure 15). During the roller application, the sealer filled the empty spaces between the individual fibers, creating a layer of uneven thickness. This led to irregularities in the form of randomly occurring elevations and depressions. Such a structure further influenced the subsequent layer applied and the resulting degree of surface unevenness.

**Figure 16:**
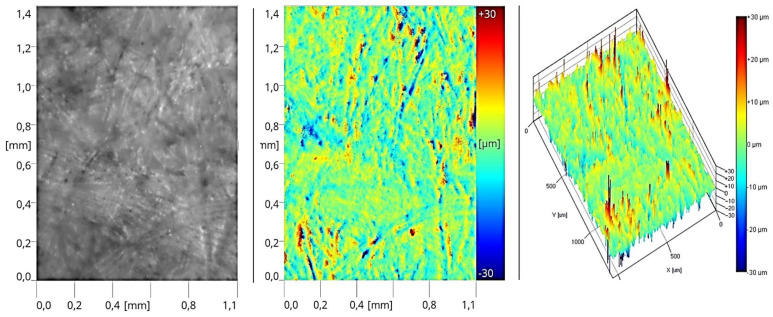
Surface image of the coating formed in Stage II (two layers of sealer).

After applying the second layer of sealer at half the previous application rate (Stage II of the process), a similar microstructure of the coating was observed. The formed surface also displayed an imprint of the HDF board’s structure, though it was less pronounced. In this case, the roughness profile is significantly lower compared to the coatings made with sealers (Figure 16).

**Figure 17:**
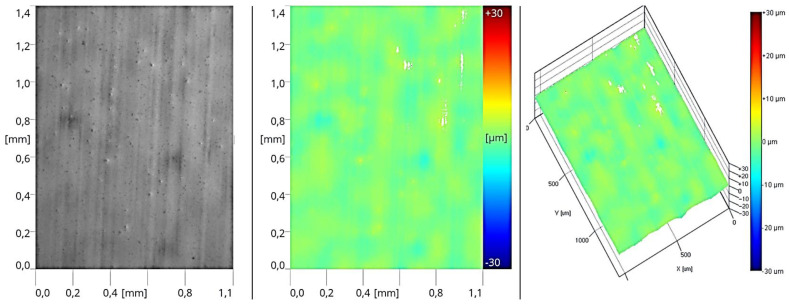
Surface image of the coating formed in Stage III (two layers of sealer + one layer of basecoat).

The above operations enabled the creation of an appropriate roughness profile on the surface of the sealers, facilitating proper adhesion for subsequent layers of coating products. In the next stage, the formed layer of primer reflected the surface of the coating obtained in the second stage of the process after intermediate sanding (Figure 17). This was due to the application of 12 g/m^2^ of the coating product, resulting in a thin cured layer. In this case, the roughness profile is significantly lower compared to the sealer coatings.

**Figure 18:**
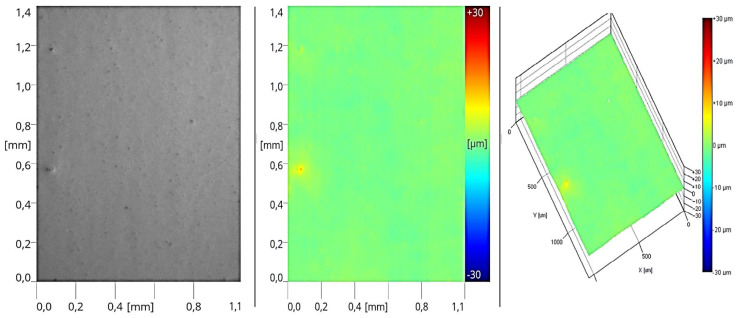
Surface image of the coating formed in Stage IV (two layers of sealer + two layers of basecoat).

The application of the second layer of the basecoat resulted in a uniform coating and a reduction in surface roughness (Figure 18).

**Figure 19:**
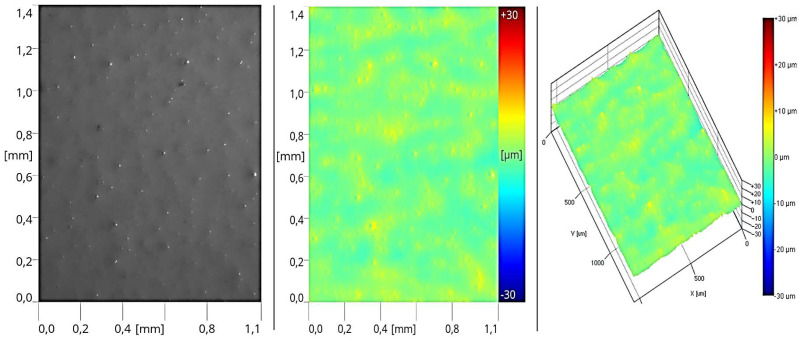
Surface image of the coating formed in Stage V (two layers of sealer + two layers of basecoat + one layer of topcoat).

In the final stage, after applying a 5 g/m^2^ layer of matte topcoat, changes in the topography and surface roughness were observed (Figure 19).

The image revealed inclusions in the form of matting agent particles, which surfaced during curing, as well as degassing air bubbles from the foamed lacquer material. This disrupted the morphology of the coating, providing the appropriate surface roughness to achieve light diffusion and, consequently, a low gloss finish. Additionally, the thin coating thickness facilitated this process.

The surface topography images obtained in this study also revealed the presence of different microstructures in the applied layers at various stages of production [44]. Immediately after the application of the sealer layer, the surface structure of the HDF board was reflected, which became progressively smoother with each additional layer of products. An increased application of the basecoat layer effectively reduced surface imperfections left after the filling process (by sealers) and ensured better uniformity and smoothness of the substrate. However, in the final stage, after the application of the topcoat, slight irregularities appeared.

### 3.5. Energy Consumption Analysis

**Figure 20:**
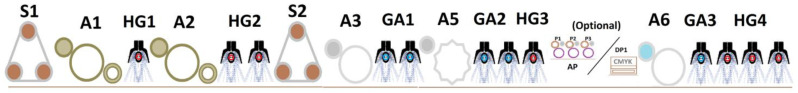
Selected schematic diagram for comparison with a selected process. Process description: (**A1**) sealer 1–50 g/m^2^; (**A2**) sealer 2–25 g/m^2^; (**A3**) basecoat 1–12 g/m^2^; (**A5**) basecoat 2–30 g/m^2^; (**A6**) topcoat 1–5 g/m^2^; (**S1**,**S2**) sanding; (**AP**: **P1**,**P2**,**P3**) analog printing machine; (**DP1**) digital printing machine; (**GA1**,**GA2**,**GA3**) gallium lamp 120 W/cm; (**HG1**,**HG2**,**HG3**,**HG4**) mercury lamp 120 W/cm.

The comparison of energy consumption for the curing processes of selected systems conducted during this study was made in relation to the standard production line, which uses mercury emitters and mercury emitters with added gallium, as shown in Figure 20.

For the electromagnetic radiation curing process, three sections were distinguished:

- Section 1: Curing of the surface after applying sealer layers (Stage I; Stage II);
- Section 2: Curing of the surface after applying the primer layer (Stage III; Stage IV);
- Section 3: Curing of the surface after applying the topcoat layer (Stage V).

The data were correlated with the product being manufactured at that time. Knowing the number of coated samples, the amount of energy delivered to the material per unit area (Wh/m^2^—watt-hours per square meter of surface) was calculated.

**Figure 21:**
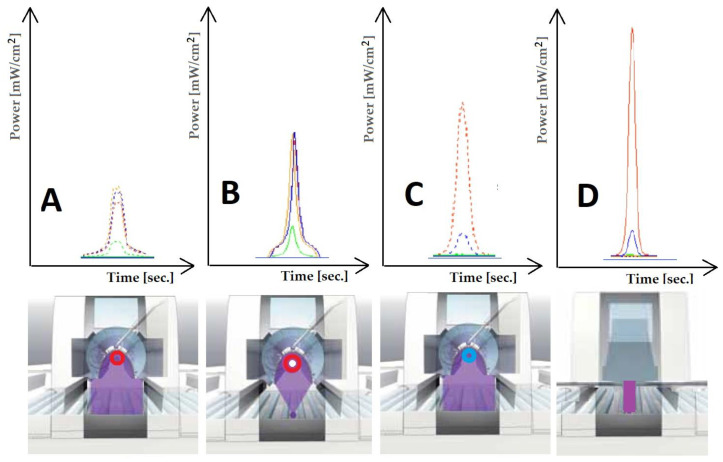
Differences in the intensity and distribution of UV emitter. Description: (**A**) mercury lamp 120 W/cm; (**B**) super-focus mercury lamp 120 W/cm; (**C**) gallium lamp 120 W/cm; (**D**) LED lamp 395 nm 12 W/cm^2^.

The differences in the UV emitters used are illustrated in Figure 21.

The obtained data were then correlated with the product being manufactured at that time. Knowing the number of produced items, the amount of energy delivered to the material per unit area (Wh/m^2^) was calculated. The results are presented in Table 6.

## 4. Conclusions

Based on the results of the conducted research, the following was determined:

1. At all stages of the technological process, a decrease in the wetting angle was observed with a reduction in energy density. This effect was most noticeable in the initial stage, where the highest amounts of lacquer product were applied during the entire printing process and its preliminary pre-gelation.
2. Significant differences between the angles (CA and CAc) were observed in the case of coatings formed from sealers (Stages I and II). The greatest discrepancies were noted with the lowest doses of UVA and UVV 26 mJ/cm^2^ for system no. 6, reaching up to 30 degrees. This confirms the need to consider the Wenzel correction factor when evaluating the wettability of HDF boards.
3. As energy density decreases, the roughness of the coatings increases. The research did not reveal a clear trend in changes in the Ra and Rz parameter values depending on the measurement direction.
4. Surface topography studies are useful for analyzing the finishing technology of HDF boards with UV coatings cured by Hg and LED lamps.
5. The greatest energy savings, reaching up to 50%, were observed in Stages III and IV of the technological process, as a result of the application of UV LED emitters and modules focusing on electromagnetic radiation.

## Figures and Tables

**Table 1:** Properties of UV varnish products.

	Name of UV Varnish Products				
Properties	IQ-UV 03040 Sealer	IQ-UVC03284 Basecoat	IQ-UVC03285 Basecoat	IQ-HYC02486 Ink	IQ-HYC02806 Topcoat
Polymer base	acrylic	acrylic	acrylic	waterborne	acrylic
Color	colorless	white	white	black	white
Solid content [%]	95.3 ± 3	97.6 ± 3	97.5 ± 3	20 ± 3	97.5 ± 3
Viscosity of delivery	65–85	90–140	35–50	20–35	30–40
	(flow cup 8 mm)	(flow cup 6 mm)	(flow cup 8 mm)	(flow cup 4 mm)	(flow cup 6 mm)
Processing temperature [°C]	Between 20–50				

**Table 2:** Preparation of the test substrate.

Samples	Speed [m/min]	Amount for Layer [g/m^2^]	Type	Power UVA [mW/cm^2^]	Power UVV [mW/cm^2^]	Energy UVA [mJ/cm^2^]	Energy UVV [mJ/cm^2^]	Power LED395 [mW/cm^2^]	Energy LED395 [mJ/cm^2^]
Process sheme (Stage I)	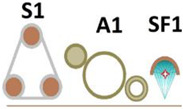								
3	40	50	sealer	1523	1421	115	112	-	-
4	40	50	sealer	1155	1066	89	86	-	-
5	40	50	sealer	761	711	57	56	-	
6	40	50	sealer	385	369	26	26	-	-
Process sheme (Stage II)	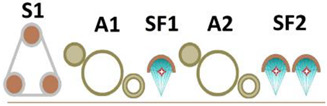								
7	40	25	sealer	2385	2211	171	165	-	-
9	40	25	sealer	1640	1538	113	110	-	-
10	40	25	sealer	1238	1136	89	85	-	-
12	40	25	sealer	363	346	27	26	-	-
Process sheme (Stage III)	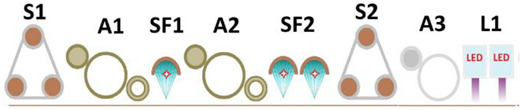								
26	40	12	basecoat 1	-	-	-	-	10,420	534
27	40	12	basecoat 1	-	-	-	-	8617	444
29	40	12	basecoat 1	-	-	-	-	6721	346
30	40	12	basecoat 1	-	-	-	-	5501	289
Process sheme (Stage IV)	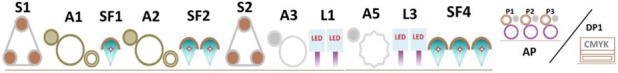								
36	40	30	basecoat 2	2865	2742	304	303	9327	513
37	40	30	basecoat 2	1487	1428	150	150	9327	513
42	40	30	basecoat 2	827	797	91	92	9327	513
43	40	30	basecoat 2	677	676	77	80	6721	346
Process sheme (Stage V)	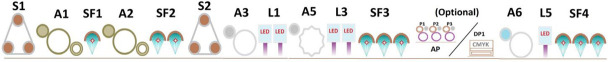								
75	40	5	topcoat	3560	3397	400	398	5501	289
77	40	5	topcoat	2865	2742	304	303	5501	289
79	40	5	topcoat	960	931	104	105	5501	289
81	40	5	topcoat	364	361	34	32	5501	289

**Table 3:** Measurement system’s parameters [41].

Method	Fringe Projection Phase Shifting
XY pixel size:	1.1 μm × 1.1 μm
Measured range in Z direction	1 μm–60 μm
Lateral sampling (XY):	1.41 mm × 1.06 mm
Measurement speed	5–30 s (1280 × 960 measurement points)
Imaging options	Optical image, 2D and 3D roughness graphs
Method:	Fringe projection phase shifting
XY pixel size:	1.1 μm × 1.1 μm
Measured range in Z direction	1 μm–60 μm
Lateral sampling (XY):	1.41 mm × 1.06 mm

**Table 4:** Measurement system parameters.

Detachment Type	Detachment Occurring in a Given System
A	Cohesive in a substrate
A/B	Adhesive between a substrate and the first sealer
B	Cohesive in the first sealer
B/C	Adhesive between the first sealer and second sealer
C	Cohesive in the second sealer
C/D	Adhesive between the second sealer and first basecoat
D	Cohesive in the first basecoat
D/E	Adhesive between the first basecoat and second basecoat
E	Cohesive in the second basecoat
E/PRINTING	Adhesive between the second basecoat and printing
PRINTING	Cohesive in the printing
PRINTING/F	Adhesive between the printing and topcoat
F	Cohesive in the topcoat

**Table 5:** Type of delamination occurring in the tested variants after adhesion strength test.

Samples	Speed [m/min]	Amount for Last Layer [g/m^2^]	Type	Evaluation of Adhesion [%]				
3	40	50	sealer	80A, 20A/B	100A	100A	100A	100A
4	40	50	sealer	90A, 10A/B	100A	100A	100A	80A, 20A/B
5	40	50	sealer	60A, 40A/B	25A, 75A/B	40A, 60A/B	60A, 40A/B	40A, 60A/B
6	40	50	sealer	80A, 20A/B	70A, 30A/B	30A, 70A/B	40A, 60A/B	20A, 80A/B
7	40	25	sealer	100A	100A	95A, 5A/B	100A	100A
9	40	25	sealer	100A	100A	100A	95A, 5A/B	90A, 5B, 5A/B
10	40	25	sealer	80A, 20C	85A, 15C	90A, 10C	85A, 15C	85A, 15C
12	40	25	sealer	70A, 30C	85A, 5C, 10B/C	85A, 10C, 5B/C	70A, 30C	95A, 5B/C
26	40	12	basecoat 1	100A	100A	90A, 10A/B	70A, 30A/B	100A
27	40	12	basecoat 1	100A	90A, 10A/B	100A	70A, 30A/B	100A
29	40	12	basecoat 1	70A,30A/B	50A, 50A/B	100A	90A, 10A/B	100A
30	40	12	basecoat 1	20A, 80C/D	100C/D	15A, 85C/D	15A, 85C/D	25A, 75C/D
36	40	30	basecoat 2	100E/Printing	100E/Printing	100E/Printing	100E/Printing	100E/Printing
37	40	30	basecoat 2	100E/Printing	100A	10A, 90E/Printing	20A, 80E/Printing	100A
42	40	30	basecoat 2	70A, 30E/AP	100A	90A, 10A/B	100A	100A
43	40	30	basecoat 2	70F, 20A, 10A/B	70A, 30D/E	100A	100A	5A, 95D/E
75	40	5	topcoat	90A, 10A/B	100A	100A	90A, 10A/B	100A
77	40	5	topcoat	100A	100A	80A, 20A/B	100A	80A, 20A/B
79	40	5	topcoat	100A	100A	100A	90A, 10A/B	100A
81	40	5	topcoat	100A	80A, 20Print/F	80A, 20Print/F	100A	100A

**Table 6:** Electrical energy allocated for curing coating products in a given section.

Measurement Section	Section 1	Section 2	Section 3
Stage	Step I, Step II	Step III, Step IV	Step V
Process description	Curing of sealer layers	Curing of basecoat layers	Curing of topcoat layer
Average energy values standard line [Wh/m^2^]	34.67	50.32	58.6
Average energy values for research line [Wh/m^2^]	23.44	25.46	43.8
Energy Savings [%]	32	50	25

## Data Availability

The data presented in this study are available upon request from the corresponding author. The data are not publicly available due to privacy.
